# The Forgotten Joint Score after total knee arthroplasty with a kinematic alignment‐optimized femoral component matches total hip arthroplasty

**DOI:** 10.1002/ksa.12712

**Published:** 2025-06-01

**Authors:** Stephen M. Howell, Ahmed Zabiba, Alexander J. Nedopil, Maury L. Hull

**Affiliations:** ^1^ Department of Biomedical Engineering University of California Davis California USA; ^2^ University of California at Davis School of Medicine Sacramento California USA; ^3^ Orthopädische Klinik König‐Ludwig‐Haus, Lehrstuhl für Orthopädie der Universität Würzburg Würzburg Germany; ^4^ Department of Orthopedic Surgery University of California Davis California USA; ^5^ Department of Mechanical Engineering University of California Davis California USA

**Keywords:** Forgotten Joint Score, kinematic alignment, Oxford Knee Score, patient‐reported outcomes, prosthetic trochlear groove, total knee arthroplasty

## Abstract

**Purpose:**

In kinematic alignment (KA) total knee arthroplasty (TKA), 50% of patients treated with a femoral component that has a 6° valgus prosthetic trochlear groove (PTG) show an abnormal quadriceps line of pull directed laterally to the groove. Lateral misalignment is reported to decrease the Forgotten Joint Score (FJS) by 17–24 points. Therefore, this study aimed to determine whether using a femoral component with a 20° valgus PTG, which minimizes the risk of lateral misalignment, can achieve a mean FJS that meets the 70‐point threshold for a successful outcome in total hip arthroplasty (THA) and the mean value of 67 points for knees in the U.S. population.

**Methods:**

The study analyzed the first 127 patients who underwent KA TKA with the 20° valgus KA‐optimized femoral component. At a minimum 2‐year follow‐up, patients were sent an online survey to complete the FJS and Oxford Knee Score (OKS) to assess function and report instances of revision knee surgery. Ten of the 127 cases were excluded: two required revision surgery. Four patients did not complete the questionnaire but reported no reoperations. The families of two individuals who passed away provided the same response. Two patients could not be contacted.

**Results:**

The analysis focused on 117 KA TKAs, with a mean FJS of 75 points, 5 points higher than the 70‐point threshold considered successful for THA and exceeding the normative value of 67 points for knees. The mean OKS was 43 points.

**Conclusion:**

Primary KA TKA with a 20° valgus PTG can achieve an FJS comparable to THA with high functionality, as shown by 70% and 25% of subjects receiving an excellent (48–42) or good (41–34) OKS.

**Level of Evidence:**

Level III.

**Abbreviations**
FJS: Forgotten Joint Score
KA: kinematic alignment
KA TKA: kinematically aligned total knee arthroplasty
SD: standard deviation
TKA: total knee arthroplasty

## INTRODUCTION

The Forgotten Joint Score (FJS) is an important patient‐reported outcome measure for assessing individuals who undergo total knee arthroplasty (TKA) and total hip arthroplasty (THA). This score has a maximum value of 100 points and is based on a 12‐item questionnaire that evaluates a patient's subjective ability to forget about their joint arthroplasty [5, 41].

Generally, patients with a TKA report a lower FJS than THA patients [41]. For TKA, a successful FJS should be at least 70 points, as it aligns with the threshold set for THA and 67 for the knee in the US general population [9, 36].

Implementing strategies to enhance the FJS for patients undergoing TKA is essential. Randomized controlled trials and two case series comparing patients with a kinematically aligned (KA) TKA in one knee and a mechanically aligned (MA) TKA in the other reported FJS scores 3, 7 and 15 points higher on the side treated with KA [38, 40, 42].

Another factor, besides KA, is the conformity of the tibiofemoral joint. KA TKA with a medial ball‐in‐socket and a lateral flat articular conformity had a 16‐point higher FJS than a PCL‐retaining implant [8], and a 10‐point advantage over a PCL‐substituting implant [39].

**Figure 1:**
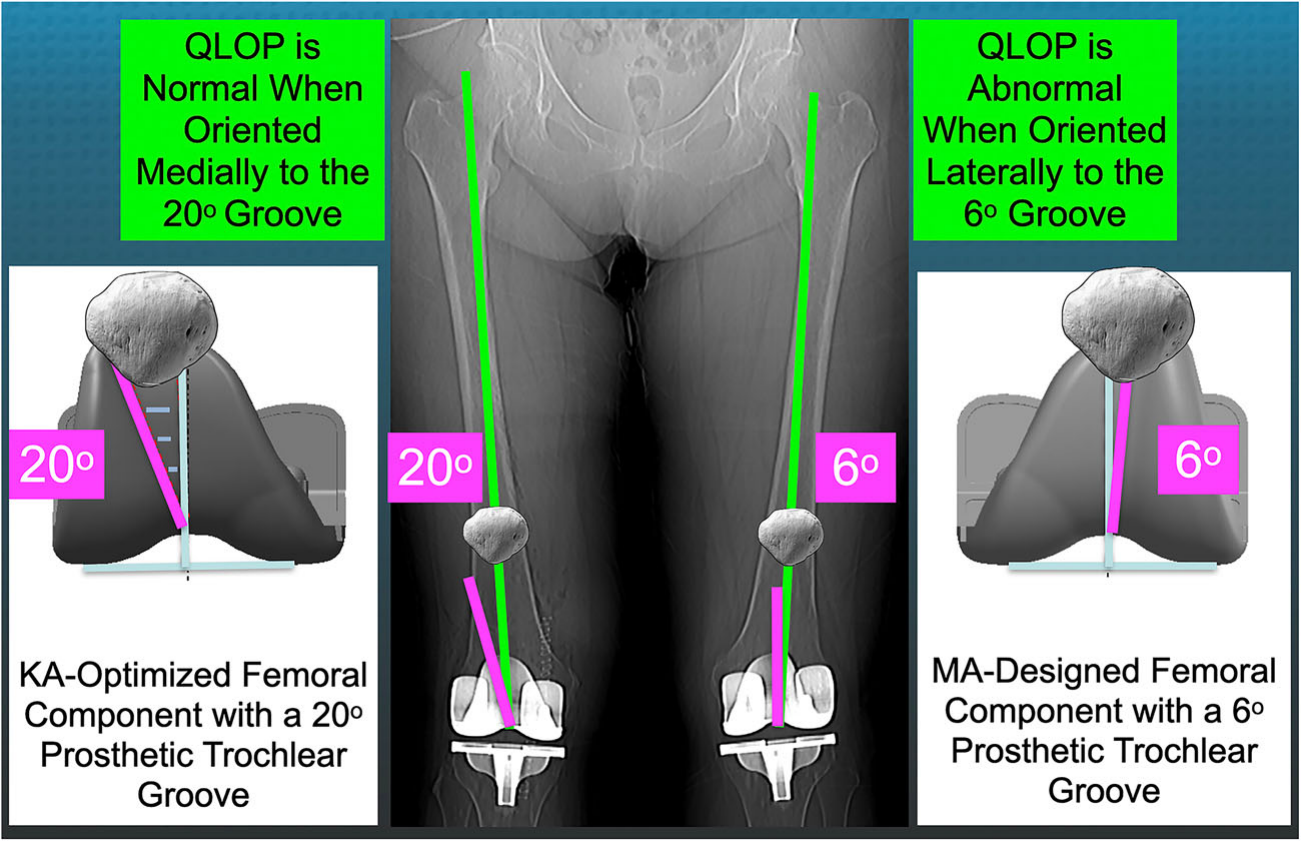
The composite includes schematics of the KA‐optimized femoral component, featuring a 20° prosthetic trochlear groove (left) and the MA‐designed femoral component with a 6° groove (right), along with a radiograph (centre) of a patient with bilateral KA TKAs that incorporate each femoral component. The quadriceps line of pull (QLOP) is oriented parallel to the spherical axis, which connects the centre of the femoral head and medial femoral condyle [23]. The QLOP should maintain a medial orientation relative to the prosthetic trochlear groove to facilitate native patella tracking. A lateral orientation is deemed abnormal and is associated with a 17‐ to 24‐point lower Forgotten Joint Score, which occurs in over half of KA TKAs with a 6° groove [16, 18]. KA, kinematic alignment; TKA, total knee arthroplasty.

Finally, the valgus angle of the femoral component's prosthetic trochlear groove (PTG) can significantly influence the FJS (Figure 1). In more than half of the patients with a 6° valgus PTG, the quadriceps line of pull (QLOP) was misaligned laterally to the groove. Patients whose QLOP was within or medial to the groove recorded FJS scores 17–24 points higher [16, 18]. Increasing the valgus angle to at least 17° may enhance the FJS, especially for specific types of coronal plane alignments of the knee (CPAK) [18].

In November 2021, a femoral component with a 20° valgus PTG became available. This study evaluated the first 127 patients treated with this KA‐optimized femoral component. The objectives were twofold: first, to determine whether the FJS meets the established threshold of 70 points for a successful THA and whether it exceeds the normative value of 67 points for the knee; second, to assess functionality by recording the Oxford Knee Score (OKS). Additionally, the study examined preoperative factors to identify potential reasons for lower scores in some patients and to investigate the types of knee revisions performed.

## METHODS AND MATERIALS

The study analyzed the first 127 patients who underwent KA TKA with the 20° valgus KA‐optimized femoral component. Each patient met the following criteria before undergoing KA TKA: fulfilled the Centers for Medicare & Medicaid Services guidelines for medical necessity for TKA and presented with Kellgren–Lawrence Grades III to IV osteoarthritis. Patients with any severity of knee varus or valgus deformity or any degree of flexion contracture were treated. The reasons for excluding 10 patients are outlined in Table 1.

**Table 1:** The table lists the reasons two patients required reoperation and the status of eight patients with incomplete 2‐year follow‐up among 127 consecutive KA TKAs treated with the KA‐optimized femoral component.

Patient	Age and sex	TKA reoperation (Yes/No)	Month and value of last Forgotten Joint Score
1	71 y/o Male	Reoperation at 2 months for deep infection, successful two‐stage revision	34 months, 75 points
2	65 y/o Female	Reoperation at 20 months for cosmetic malrotation of ankle	16 months, 43 points
3	93 y/o Female	Died at 28 months, no reoperation per daughter	19 months, 44 points
4	69 y/o Male	Died at 14 months, no reoperation per wife	7 months, 83 points
5	81 y/o Female	Spoke with patient at 34 months	12 months, 15 points
6	64 y/o Female	Spoke with patient at 25 months	4 months, 8 points
7	61 y/o Female	Spoke with patient at 27 months	3 months, 0 points
8	92 y/o Female	Spoke with patient at 27 months	1 month, 27 points
9	69 y/o Female	Lost to follow‐up, EMR indicated no reoperation at 34 months	4 months, 19 points
10	70 y/o Female	Lost to follow‐up	2 months, 8 points

Abbreviation: EMR, electronic medical record; KA, kinematic alignment; TKA, total knee arthroplasty.

On the day of the initial consultation, but before seeing the surgeon, each patient filled out the OKS (48 best, 0 worst) and Knee Injury and Osteoarthritis Outcome Score for Joint Replacement (KOOS, JR) (100 best, 0 worst) and provided patient demographics on an iPad. In addition, the physician assistant measured the knee's extension, flexion, and alignment deformity with a long‐arm goniometer and recorded it on the iPad. All values were synced to an online patient data registry (CareSense, https://www.caresense.com/, accessed on 29 January 2025).

In November 2021, the surgeon began receiving a limited inventory of a new cemented KA‐optimized femoral component with a 20° valgus PTG (GMK SpheriKA, Medacta International, www.medacta.com, accessed on 29 January 2025). Patients underwent KA TKA with the KA‐optimized femoral component when the intraoperative inventory had the patient's correct size.

The surgeon performed the KA TKA with PCL retention through a mid‐vastus approach using manual instruments with a reported accuracy greater than robotics and a negligible learning curve for the inexperienced surgeon [15, 27]. The details of the three balancing steps, which are gap‐balancing the tibial resection to restore a tight rectangular space with a spacer block in extension, restoring the pre‐arthritic medial tibial slope, and selecting the optimal insert thickness with an insert goniometer, are previously described [26, 28]. Calliper measurements of the bone resections, which confirm the femoral and tibial components resurface the pre‐arthritic knee, were recorded intraoperatively on a verification sheet and in the operative note. These measurements verify that the setting of the components met the criteria of KA and provide a quantitative record that the components restored the pre‐arthritic joint lines within 0.5 mm, which is an accuracy necessary to optimize post‐operative OKS and FJS [35].

**Figure 2:**
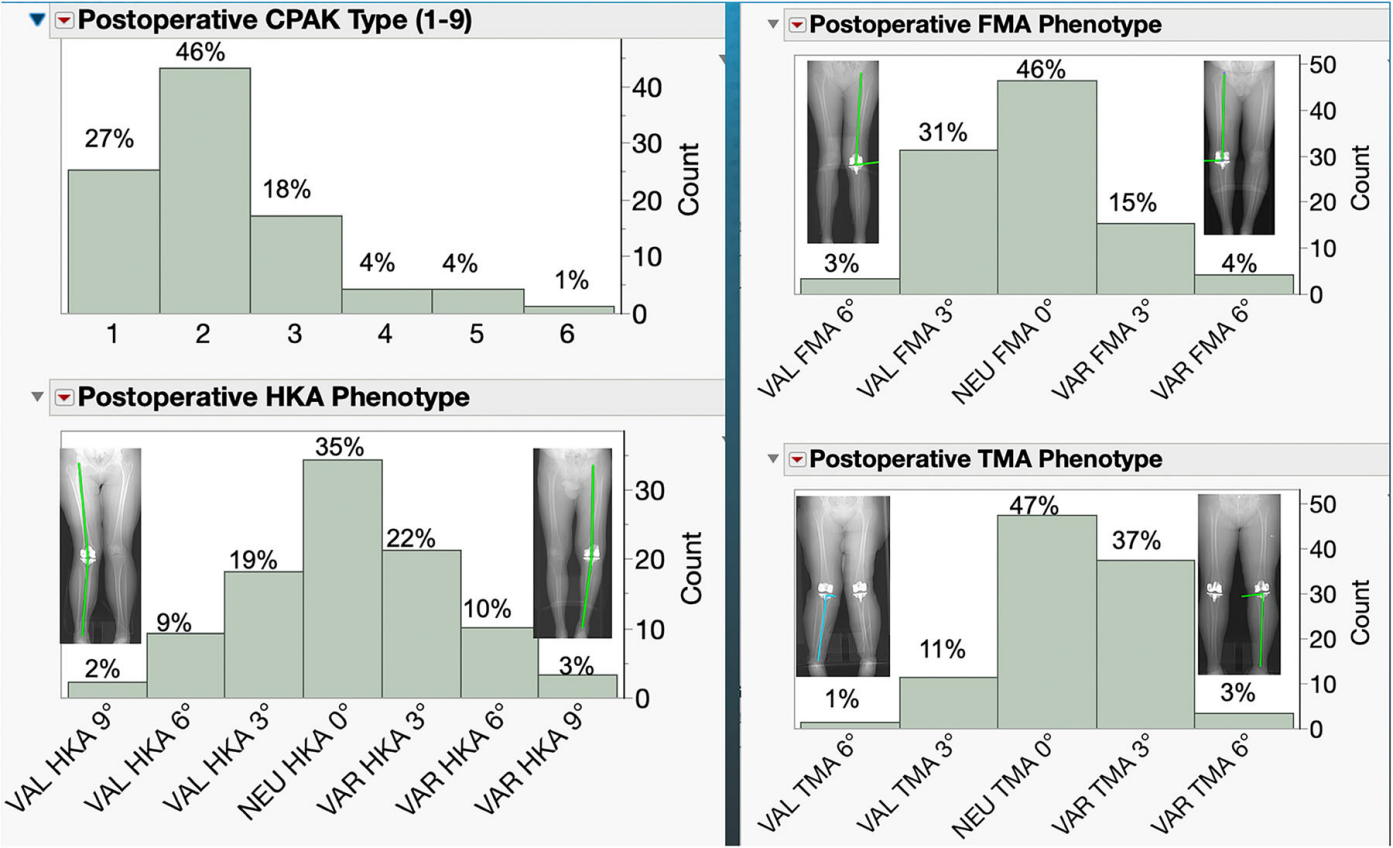
The distributions illustrate the post‐operative percentages of KA TKAs categorized within CPAK type and by HKA, FMA and TMA functional phenotypes according to ranges reported for normal limbs [10, 11, 12, 22]. The CT long‐leg scanogram above each column indicates the most aberrant alignment, which occurred in no more than 7%–4% [11]. However, according to the principles of KA, the alignment of the components is correct and not aberrant, as the components were set to resurface the pre‐arthritic knee and are within 2° of the contralateral side [30]. CPAK, coronal plane alignment of the knee; CT, computed tomography; FMA, femoral mechanical angle; HKA, hip–knee–ankle angle; KA, kinematic alignment; TKA, total knee arthroplasty; TMA, tibial mechanical angle.

On the day of discharge, each patient underwent an A‐P, non‐weight‐bearing, long‐leg scanogram of both legs performed by a CT scanner with a radiation dose of 0.5 mSv, less than a long‐leg radiograph [18, 24]. Ninety‐nine out of 117 patients had a correctly oriented limb for measurement without evidence of hip or ankle arthroplasty or femoral and tibial malunion. The following parameters were measured and subsequently reported in distributions: Coronal plane alignment of the knee (CPAK), the hip–knee–ankle angle, femoral mechanical angle and tibial mechanical angle functional phenotypes using reported protocols [10, 12, 22] (Figure 2).

All patients followed a protocol designed to safely discharge individuals aged 50–89 from the hospital on the day of surgery. The recovery plan emphasized exercises that patients could perform without the guidance of a physical therapist [1].

After a minimum follow‐up of 24 months, the first 127 patients treated with the 20° KA‐optimized femoral component received a text message and email link to an online survey to complete the FJS and OKS and report any instances of revision knee surgery.

An institutional review board (Advarra CIRIBA) provided an exempt determination (Pro00084801) for a retrospective analysis of de‐identified patient data obtained from a prospectively archived records database.

### Data analysis

An a priori sample size calculation was conducted using a one‐sided Mann–Whitney *U* test to evaluate non‐inferiority. The non‐inferiority margin for the FJS was established based on the minimally clinically important difference of 14 points for a cohort of patients [5]. With a significance level of *α* = 0.05, a statistical power of 0.80, a standard deviation (SD) of 25, and assuming equal sample sizes in both groups, the analysis determined that a minimum of 44 patients was required to assess whether the FJS following KA TKA reached the THA success threshold of 70 points at the 2‐year mark [36]. As a result, analyzing data from 117 subjects provided adequate power for the study.

Statistical software was used to calculate the mean and SD of the FJS and OKS (JMP Pro, version 18.0.1, https://www.jmp.com, accessed on 29 January 2025). To assess whether preoperative patient demographics and characteristics could predict a lower FJS, differences in these factors were analyzed among three cohorts of patients: one cohort with a 2‐year FJS of 70 points or greater, a second cohort with scores between 40 and 70 points, and a third cohort with scores below 40 points. Analyses were conducted using the Kruskal–Wallis test and chi‐square analysis.

## RESULTS

**Figure 3:**
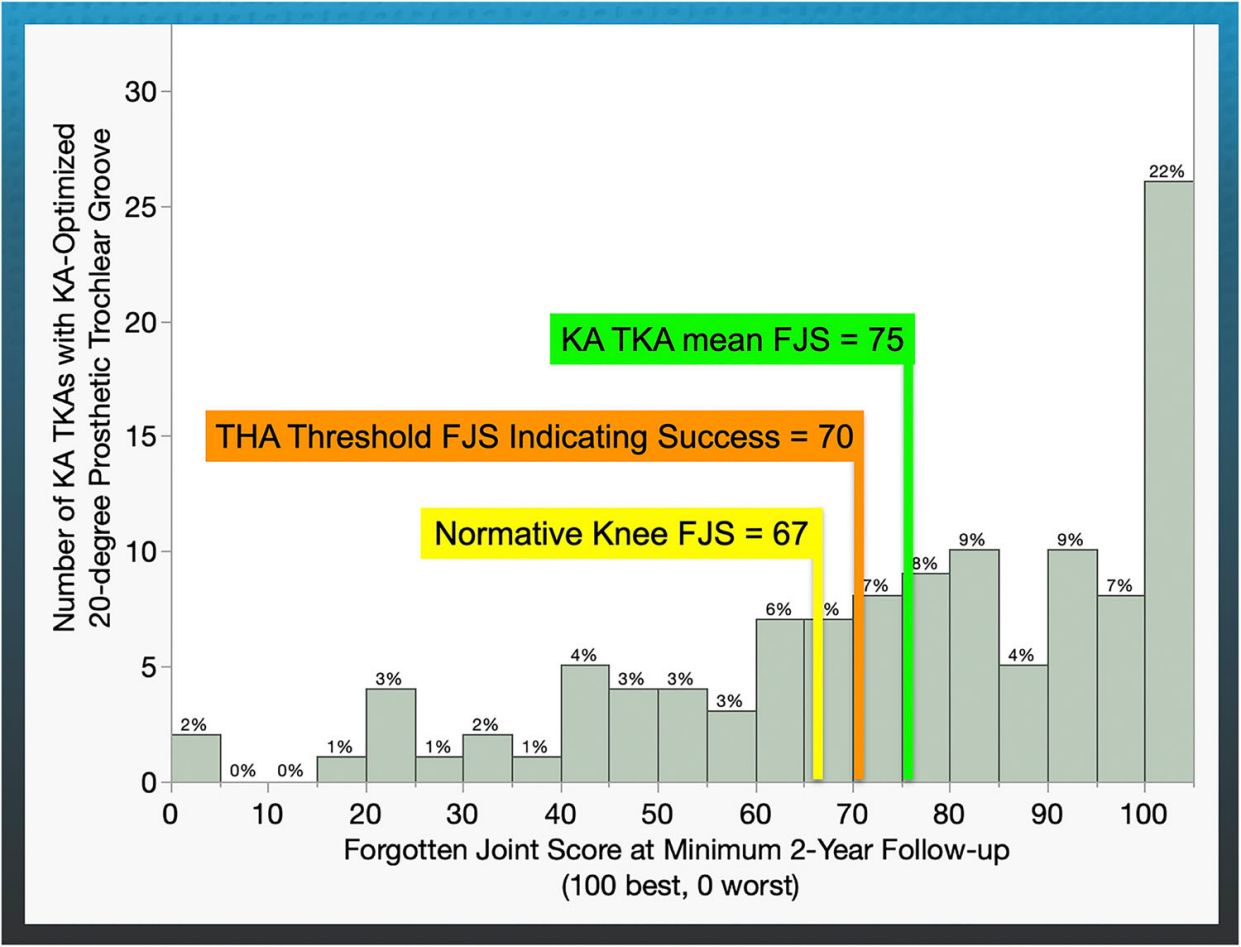
The distribution illustrates the Forgotten Joint Score (FJS) at a minimum follow‐up of 2 years (*x*‐axis), the number of KA TKAs with a KA‐optimized 20° prosthetic trochlear groove (*y*‐axis), and the percentage of patients in each 5‐point bin. The mean FJS of 75 points of the KA TKAs exceeded the 70‐point threshold for successful total hip arthroplasty (THA) and the 67‐point mean normative value for knees in the general population of the United States [9, 36]. KA, kinematic alignment; TKA, total knee arthroplasty.

Consequently, the analysis focused on 117 KA TKAs, with an average age of 68 years, including 65 women (Table 2). The mean FJS at a minimum of 2 years was 75 ± 25 points for the KA TKAs, which exceeds the 70‐point threshold for successful THA and the 67‐point average normative value for knees in the general US population (see Figure 3). The mean OKS was 43 ± 6, with 70% of subjects reporting an excellent score of 42–48, 25% a good score from 34 to 41, 2.5% a fair score of 27–33, and 2.5% a poor score according to the Kalairajah classification [25]. The three cohorts with high, fair, and poor FJS showed no significant differences in preoperative patient demographics and characteristics (Table 3).

**Table 2:** The table presents values for the preoperative demographics and clinical characteristics of 117 patients who reported the Forgotten Joint Score at a minimum follow‐up of 2 years.

Preoperative demographics and clinical characteristics	Number of patients	Mean ± SD
Age	*N* = 117	68 ± 8.9
Sex	Males *N* = 52, Females *N* = 65	
Body mass index (kg/m^2^)	N = 115	30 ± 5.9
Knee Injury and Osteoarthritis Outcome Score	N = 111	51 ± 14.4
Oxford Knee Score	N = 114	25 ± 8.7
Knee extension (°)	N = 114	8.8 ± 7.1
Knee flexion (°)	N = 114	118 ± 9.6
Range of motion (°)	N = 113	109 ± 13.7
Radiographically determined osteoarthritic knee deformity	Varus *N* = 73 (62%), Valgus *N* = 35 (30%), Patellofemoral *N* = 9 (8%)	

Abbreviation: SD, standard deviation.

**Table 3:** The table displays the number of patients, preoperative demographics and clinical characteristics, categorized into three cohorts based on their Forgotten Joint Score at a minimum 2‐year follow‐up.

Preoperative demographics and clinical characteristics	76 Patients with Forgotten Joint Score of 70 or greater	30 Patients with Forgotten Joint Score of 40–70	11 Patients with Forgotten Joint Score of Less than 40	Significance
Age	68 ± 9	67 ± 9	69 ± 10	NS, *p* = 0.7944
Sex	Males *N* = 33, Females *N* = 43	Males *N* = 18, Females *N* = 12	Males *N* = 1, Females *N* = 10	*p* = 0.014
Body mass index (kg/m^2^)	29 ± 6	30 ± 6	31 ± 5	NS, *p* = 0.5966
Knee Injury and Osteoarthritis Outcome Score	53 ± 15	48 ± 13	47 ± 12	NS, *p* = 0.2425
Oxford Knee Score	26 ± 9	23 ± 9	24 ± 8	NS, *p* = 0.2089
Knee extension (°)	8 ± 7	10 ± 7	9 ± 9	NS, *p* = 0.5227
Knee flexion (°)	119 ± 9	119 ± 10	115 ± 12	NS, *p* = 0.4994
Range of motion (°)	110 ± 12	109 ± 16	106 ± 20	NS, *p* = 0.6265
Radiographic osteoarthritic knee deformity	Varus 44, valgus 27, patellofemoral 5	Varus 20, valgus 7, patellofemoral 3	Varus 9, valgus 1, patellofemoral 1	NS, *p* = 0.3813

Abbreviation: NS, not significant.

## DISCUSSION

The most important finding of this study is that primary TKA can achieve an FJS comparable to THA with high functionality, as shown by 70% and 25% of subjects receiving an excellent (48–42) or good (41–34) OKS. Factors that enhance the FJS to varying degrees include KA, a 20° valgus PTG, and an implant featuring a medial ball‐in‐socket and a lateral flat insert articulation. Secondary findings revealed that preoperative patient demographics and characteristics did not account for patients with a lower FJS, indicating that these factors cannot assist in predicting the expected FJS.

Several MA TKA studies have reported that the mean FJS of a cohort of TKA patients met or exceeded the 75‐point threshold for a successful THA; however, the follow‐up methods had shortcomings that potentially explain the high score. One study reported a mean FJS of 76 at 2 years; however, none of the FJS were patient‐reported, as they were collected through phone interviews, which are known for interviewer bias. Additionally, only a subset of 100 surveys was obtained from a screening of a database of 2315 knee arthroplasties [4]. Another study reported a mean FJS of 71 at 1 year, which did not change from 6 months. However, the 63 subjects recruited at a local orthopaedic hospital did not represent a consecutive series; 5 were lost to follow‐up [13].

Another exception is a case‐matched study of 100 MA TKA and KA TKA using a symmetric low‐conforming PCL‐substituting implant with a 6° valgus PTG. After 2 years, the study reported mean FJS scores of 78 for MA TKA and 92 for KA TKA [7]. The notably high FJS score of 92 for KA TKA is challenging to reconcile, as it significantly exceeds all previously reported scores for this type of procedure. For instance, a Level I trial involving KA TKA that randomized implant designs found that the group receiving a symmetric low‐conforming PCL‐substituting implant with a 6° valgus PTG had a mean FJS of only 58, which is 34 points lower than the recent study's findings. The KA cohort that used a medial ball‐in‐socket and a lateral flat articular implant with a 6° valgus PTG achieved a mean FJS of 68, closer to the 75‐point FJS observed in our study [39]. One possible explanation for the 7‐point higher FJS in our current study could be the reduced risk of a misaligned QLOP achieved by using a 20° valgus PTG, which has the potential to enhance the FJS by 17 points in more than half of the patients in whom a 6° valgus PTG groove is deemed too medial [16, 18, 37].

It is unclear why 35% (41 out of 117) of patients reported an FJS below the 70‐point threshold for a successful THA and why 9% (11 out of 117) recorded a score below 40. A study of 585 MA TKAs used regression and cluster analysis and reported lower FJS for patients with a higher body mass index (BMI), lower age and women [2]. The present study's preoperative patient demographics and characteristics did not explain those with a lower FJS. Only three identifiable causes for scores below 40 points emerged from discussions with the eleven patients: symptomatic hip arthritis on the side of the KA TKA, neuropathy, and dementia.

The findings of a high FJS and OKS after KA TKA align with 3D modelling studies, indicating that the patient's native trochlea is better restored using the KA approach than mechanical alignment [19, 20, 29, 34]. Femoral components aligned in KA demonstrated greater biomimetic qualities than MA regarding trochlear sulcus orientation and trochlear height restoration, particularly in valgus femora [3]. The 20° valgus PTG can potentially reduce the risk of patellofemoral complications by lateralizing and opening the groove, thus preventing the medialization of the patella across a wide range of lateral distal femoral angles [17]. A recent radiologic study of sequential bilateral KA TKA in 36 patients, featuring a 20° KA‐optimized femoral component in one knee and a 6° MA‐designed femoral component in the other, confirmed that the 20° groove remained lateral to the quadriceps' line of pull in all knees [37].

The study has several limitations. One is that it reflects the experiences of only one surgeon; to enable generalization, the findings should be validated by others. A second limitation is that the reoperation rate is reported over 2 years, requiring more extensive monitoring. However, numerous studies report excellent long‐term implant survival and a low risk of tibial baseplate migration with KA TKA [6, 14, 21, 31, 32, 33]. Other factors that could predict the mean FJS were not analyzed, such as under‐ and over‐stuffing of the medial and lateral trochlear peaks. Finally, the mean FJS in this study may depend on the choice of alignment, implant design, and operative and perioperative treatment, and it could vary with other treatment conditions.

## CONCLUSION

This report on 127 consecutive patients treated with KA TKA using a new femoral component optimized for KA and featuring a 20° valgus PTG provides guidelines for counselling patients considering this treatment. Prospective patients can expect, regardless of age, BMI, sex or type of osteoarthritic knee deformity (i.e., varus, valgus and patellofemoral), that 2 years after undergoing knee arthroplasty, there is a 65% chance that their TKA will perform like a THA.

## AUTHOR CONTRIBUTIONS

All authors contributed to the study's conception, design, preparation of materials, data collection, analysis and writing. They also read and approved the final manuscript.

## CONFLICT OF INTEREST STATEMENT

Stephen M. Howell and Alexander J. Nedopil receive consulting fees and royalties from Medacta International (Castel San Pietro, Switzerland, www.medacta.com). Maury L. Hull receives research support from Medacta International (Castel San Pietro, Switzerland, www.medacta.com). The remaining author declares no conflicts of interest.

## ETHICS STATEMENT

The institutional review board approved this study, specifically the retrospective analysis of de‐identified data (Pro00084429) obtained from a prospectively archived patient records and radiographs database.

## Data Availability

The data sets used and/or analyzed during the current study are available from the corresponding author upon reasonable request.
